# Awareness, Attitudes, Needs, and Demands Toward Tooth Replacement Options Among Patients at an Emirati University‐Setting Clinic

**DOI:** 10.1155/ijod/8555753

**Published:** 2026-01-23

**Authors:** Amaweya Alsammarraie, K. Sulafa El-Samarrai, Kelvin I. Afrashtehfar, Musab Saeed, Esraa Jaber, Berksam Amr Fatouh, Shahenda A. L. Sayed, Sara Khoshniyat, Wala Alkurdi, Mawada Abdelmagied

**Affiliations:** ^1^ Department of Clinical Sciences, College of Dentistry, Ajman University, Ajman, UAE, ajman.ac.ae; ^2^ Centre of Medical and Bio-allied Health Sciences Research, Ajman University, Ajman, UAE, ajman.ac.ae; ^3^ Pedodontics and Preventive Department, University of Mashreq, Baghdad, Iraq, uom.edu.iq; ^4^ Private Practices Limited to Prosthodontics and Implant Dentistry, Abu Dhabi, UAE

**Keywords:** attitude, cross-sectional studies, dental health services, dental implants, dental prosthesis, health, oral health, patient knowledge, prosthodontics, questionnaires, tooth loss

## Abstract

**Objective:**

This study aimed to assess the awareness, attitudes, needs, and demands of patients at Ajman University regarding available tooth replacement options.

**Materials and Methods:**

A cross‐sectional survey was conducted among 200 patients attending Ajman University’s dental clinics. The sample consisted of 51.5% males and 48.5% females, all aged 18 years or older with one or more missing teeth. Participants completed a structured, closed‐ended questionnaire designed to reveal their knowledge of dental prostheses, the reasons for seeking or delaying replacement, and their preferences for prosthodontic treatment options. Statistical analysis was performed using chi‐square and Fisher’s exact tests with a significance level of *p*  < 0.05.

**Results:**

Significant associations were found between the demographic variables (independent variables) and the key prosthodontic‐related measures assessed in the study (dependent variables). Age and gender were significantly associated with the number of missing teeth (*p*  < 0.001), with older individuals and males exhibiting greater tooth loss. Education level showed a significant association with the timing of seeking tooth replacement (*p*  < 0.001). Financial constraints were the most frequently reported barrier to treatment, particularly among older and more educated participants (*p*  < 0.001). Awareness and preference for prosthodontic options were strongly influenced by education (*p*  < 0.001) and age (*p*  < 0.001), with dental implants being the most preferred option. Employment status demonstrated a significant association only with awareness of complete dentures (*p*  < 0.001), while most other dependent variables showed no significant relationship with employment status or gender.

**Conclusion:**

Patients demonstrated a high level of awareness of dental prostheses, particularly removable dentures, with implants emerging as the most preferred replacement option. Financial limitations and fear of discomfort were major barriers to seeking prosthetic treatment. Enhancing patient education and implementing financial support strategies may improve the uptake of prosthetic rehabilitation.

## 1. Introduction

The loss of natural teeth is often a distressing and functionally debilitating experience, frequently requiring extensive and costly restorative intervention [1]. Edentulism arises from multiple contributing factors, including irregular dental attendance, patient negligence, limited access to oral healthcare services, and inadequate awareness of oral health needs [1]. When missing teeth remain unreplaced, both functional and aesthetic complications may ensue, such as drifting of adjacent teeth, overeruption of opposing teeth, temporomandibular disorders, and an increased risk of additional extractions involving neighboring teeth [2]. Although many clinicians historically maintained that a complete dentition is essential for maintaining optimal masticatory function [2], patients themselves often lack awareness of the importance of timely tooth replacement and the consequences of delaying treatment [2, 3].

Edentulism has been strongly associated with socioeconomic disparities [4–6]. Previous studies have shown that patient attitudes toward prosthetic rehabilitation vary considerably and are influenced by demographic factors such as age and educational level [7–10]. Kommi et al. [11] further noted that negative attitudes may arise from repeated failures of earlier prostheses. Gender differences have also been reported, with females generally more inclined to seek early replacement of missing teeth compared to males [12–15]. Nevertheless, a substantial proportion of patients delay treatment due to financial limitations [7, 14, 16–19], fear or anxiety related to prosthodontic procedures [11], or insufficient knowledge regarding the available prosthetic options—one of the most commonly cited reasons for not replacing extracted teeth [3, 7]. Conversely, Rathi and Chhetri [16] reported that although many patients understood the consequences of nonreplacement, they preferred to seek care only when a need became noticeable.

Education plays a central role in promoting better oral and general health behaviors, and dental professionals serve as key facilitators in improving public awareness [3, 20]. Despite this, many studies have reported low levels of awareness regarding different types of prostheses [3, 21–23], while others observed higher awareness of fixed partial dentures [12] and complete dentures [15, 24]. Implant awareness remains consistently low across multiple populations [11, 15, 20, 22, 23, 25–27].

Prosthodontic rehabilitation remains one of the most common reasons for seeking dental care [27], yet evidence shows that the demand for treatment continues to fall short of the true clinical need [3, 28]. Therefore, educating partially and completely edentulous individuals about the consequences of untreated tooth loss, the indications and limitations of various prosthetic options, and the most suitable prostheses based on their individual oral condition is crucial.

Edentulism also represents a substantial global public health burden. Recent Global Burden of Disease data confirm that edentulism persists as a major oral health challenge worldwide [29]. A systematic review and meta‐analysis involving community‐dwelling adults aged 45 years and older reported a pooled global prevalence of ~22% [30]. Regionally, prevalence figures vary: in Riyadh, Saudi Arabia, a clinical cross‐sectional study of adults aged 35–74 reported a 2.6% prevalence of complete edentulism [31]; in Jeddah, a radiographic assessment of adults aged 35+ found a prevalence of 4.2% [32]; and in the United Arab Emirates, research involving care‐home residents and attendees reported that 24% were edentulous [33].

In addition to demographic characteristics, the clinical status of patients—especially the number and distribution of missing teeth and the resulting functional impairment—strongly influences awareness and demand for prosthetic rehabilitation. Recent evidence shows that individuals with fewer remaining teeth report significantly poorer oral‐health‐related quality of life and are more likely to require prosthetic treatment than those maintaining functional dentition [34]. Moreover, patterns of edentulism (e.g., extended posterior tooth loss or complete edentulism) have been shown to correlate with lower chewing ability and higher prosthetic needs, underscoring the importance of assessing clinical status alongside demographic factors [35].

According to the WHO, ~7% of adults aged 20 years or older worldwide are completely edentulous, rising to nearly 23% among those aged 60 years or above [36]. Furthermore, the 2022 WHO Global Oral Health Status Report estimates that nearly 45% of the world’s population will experience some form of oral disease—including tooth loss—during their lifetime [37]. These data underscore the substantial global burden of edentulism and oral disease and justify the need for studies assessing awareness, attitudes, and demand for prosthetic replacement in different populations. Collectively, these real‐world data highlight how important it is to assess awareness, preferences, and barriers related to tooth replacement in our local population. The present study aimed to evaluate the knowledge, attitudes, needs, and demands for tooth replacement among partially and completely edentulous patients attending Ajman University’s dental clinics, particularly focusing on their awareness of available prosthetic options and the factors preventing them from seeking treatment.

## 2. Materials and Methods

This cross‐sectional study was conducted on a convenience sample of 200 patients who attended the College of Dentistry clinics at Ajman University from February 2022 to April 2022. Ethical approval for the study was obtained from the college’s ethics committee. All participating patients provided informed consent before undergoing a clinical examination, which included an assessment of missing teeth and the presence of any artificial teeth.

Participants completed an 18‐item closed‐ended, standardized questionnaire designed specifically for this research, available in both English and Arabic. The questionnaire used in this study was specifically developed by the research team for the study objectives. A closed‐ended questionnaire is well‐suited for this study because it offers standardized response options that ensure consistency across participants, making the data easier to quantify and statistically analyze. It also reduces ambiguity, minimizes irrelevant responses, and is quick to complete, which helps improve response rates. Overall, it is an efficient and reliable tool for collecting clear, comparable data. The questionnaire was self‐administered by all participants, allowing them to respond independently. To ensure clarity and completeness, participants were provided with brief instructions and could ask the research team for clarification if needed. The inclusion criteria were patients aged 18 years or older who were partially or completely edentulous. Exclusion criteria encompassed patients missing only third molars, those with mental disabilities, individuals with language barriers, and those with careers related to dentistry. Associations were assessed between patient responses and demographic variables (age, gender, education level, employment status; Table 1), associations between these independent variables and patient responses were assessed using chi‐square and Fisher’s exact tests.

**Table 1:** Distribution of patients with missing teeth in both jaws according to demographic variables.

Variables	Upper jaw (Maxilla)		Lower jaw (Mandible)		Total	
	Mean	±SE	Mean	±SE	Mean	±SE
Age (years)						
<20	0.25	0.250	0.50	0.289	0.75	0.250
20‐29	2.29	0.625	2.14	0.516	4.43	1.091
30‐39	1.89	0.287	1.81	0.240	3.70	0.330
40‐ 49	2.66	0.446	3.02	0.448	5.68	0.803
>50	7.05	0.941	6.60	0.799	13.65	1.585
Fisher exact	13.597		14.700		17.869	
*p* value	**0.000**		**0.000**		**0.000**	
Total	3.18	0.301	3.15	0.267	6.33	0.519
Gender						
Male	4.24	0.497	3.76	0.436	8.00	0.861
Female	2.04	0.285	2.49	0.286	4.53	0.494
T test	14.221		5.748		11.750	
* p* value	**0.000**		**0.017**		**0.001**	
Education						
Graduated	3.34	0.394	3.35	0.354	6.69	0.661
Secondary school	2.63	0.562	2.78	0.548	5.40	1.087
Primary school	3.25	0.763	2.81	0.578	6.06	1.322
Employment status						
Employed	3.50	0.370	3.32	0.326	6.82	0.632
Unemployed	2.33	0.480	2.67	0.450	5.00	0.867

*Note:* Bold values represent statistically significant results (*p* < 0.05) indicating a significant association between the demographic variables and the number of missing teeth in the maxilla, mandible, or overall.

The questionnaire consisted of five domains: (1) reasons for tooth replacement, (2) past prosthodontic experience, (3) awareness of prosthesis types, (4) limitations/barriers to treatment, and (5) future prosthesis preference.

Descriptive statistics were employed for data analysis, and chi‐square and Fisher’s exact tests were conducted at a significance level of *p*  < 0.05. Participants’ demographic information, including age, gender, level of education, and employment status, was collected. Age categories were divided into five groups: less than 20 years, 20–29 years, 30–39 years, 40–49 years, and 50 years and above; educational levels were classified as primary school, secondary school, and graduated (college or higher). Employment status was noted as either employed or unemployed (Table 1).

The data collected was statistically analyzed using descriptive statistics to summarize the data. Chi‐square and Fisher’s exact tests were used to determine the significance of the relationships between the demographic variables and the responses. The significance level was set at *p*  < 0.05. The statistical analyses were performed using SPSS version 24.

## 3. Results

### 3.1. Demographic Characteristics

Analyses in this section focus on associations with demographic variables (Table 1); associations with clinical status were not assessed in the present study and are acknowledged as a limitation.

### 3.2. Timing of Seeking Tooth Replacement

Analysis showed no statistically significant association between age and the time elapsed before seeking tooth replacement after extraction (*p*  > 0.05). All age groups demonstrated similar attendance patterns, indicating that age did not influence how soon patients sought prosthetic replacement.

Females were more likely than males to seek replacement within 7–12 months after extraction (*p*  < 0.05). Patients with higher education levels also sought replacement sooner compared to those with primary or secondary education (*p*  < 0.001). Employment status did not significantly affect the timing of seeking treatment (*p*  > 0.05) (Tables 2 and 3).

**Table 2:** Distribution of patients by time of attendance following extractions, causes of not replacement according to age and gender.

Variables		Age (years)										Gender				
		<20		20–29		30–39		40–49		>50		Males			Females	
		N.	%	N.	%	N.	%	N.	%	N.	%	N.		%	N.	%
Time following Extraction (months)	0–6	1	1.92	9	17.31	15	28.85	16	30.77	11	21.15	35		67.31	17	32.69
	7–12	2	2.30	14	16.09	31	35.63	29	33.33	11	12.64	37		42.53	50	57.47
	>12	1	1.64	12	19.67	18	29.51	11	18.03	19	31.15	31		50.82	30	49.18
Chi‐square	10.506												8.017			
*p*‐Value	0.211												0.018			
Causes for not replacing teeth	1	0	0.00	15	20.55	25	34.25	17	23.29	16	21.92	35		47.95	38	52.05
	2	4	10.26	3	7.69	11	28.21	14	35.90	7	17.95	20		51.28	19	48.72
	3	0	0.00	9	30.00	7	23.33	13	43.33	1	3.33	18		60.00	12	40.00
	4	0	0.00	3	27.27	6	54.55	2	18.18	0	0.00	5		71.43	2	28.57
	5	0	0.00	4	10.00	15	37.50	9	22.50	12	30.00	4		36.36	7	63.64
Chi‐square	41.787												3.376			
*p*‐Value	0.000												0.642			
Previous prosthesis	Yes	1	1.08	14	15.05	34	36.56	27	29.03	17	18.28	43		46.24	50	53.76
	No	3	2.80	21	19.63	30	28.04	29	27.10	24	22.43	60		56.07	47	43.93
Chi‐square	2.877												1.928			
*p*‐Value	0.592												0.165			

*Note:* Causes of not replacing teeth: 1. high cost, 2. discomfort, 3. not needed, 4. fear from teeth preparation, 5. fear prosthesis coming out, and 6. time shortage.

**Table 3:** Distribution of patients by time of attendance following extractions, causes of no replacement according to education and employment status.

Variables		Education						Employment status			
		Graduated		Secondary		Primary		Employed		Unemployed	
		N.	%	N.	%	N.	%	N.	%	N.	%
The time following extraction (months)	0‐6	41	78.85	5	9.62	6	11.54	44	84.62	8	15.38
	7–12	47	54.02	17	19.54	23	26.44	60	68.97	27	31.03
	>12	39	63.93	18	29.51	4	6.56	41	67.21	20	32.79
Chi‐square	18.368							5.229			
*p*‐Value	**0.001**							0.073			
Causes for not replacing teeth	1	58	79.45	8	10.96	7	9.59	53	72.60	20	27.40
	2	21	53.85	13	33.33	5	12.82	25	64.10	14	35.90
	3	14	46.67	4	13.33	12	40.00	23	76.67	7	23.33
	4	5	71.43	2	28.57	0	.00	85.71	1	14.29	4
	5	7	63.64	2	18.18	2	18.18	7	63.64	4	36.36
	6	22	55.00	11	27.50	7	17.50	31	77.50	9	22.50
Chi‐square	24.668							3.189			
*p*‐Value	**0.003**							0.671			
Previous prosthesis	Yes	53	56.99	18	19.35	22	23.66	68	73.12	25	26.88
	No	74	69.16	22	20.56	11	10.28	77	71.96	31	28.04
Chi‐square	6.591							0.033			
*p*‐Value	**0.037**							0.855			

*Note:* Causes of not replacing teeth: 1. high cost, 2. discomfort, 3. not needed 4. fear from teeth preparation, 5. fear prosthesis coming out, and 6. time shortage. Bold values indicate significant association between the demographic variable education, and time for seeking prosthetic treatment, and whether there was a previus prosthesis or not, and with causes for not replacing teeth, employement status had no significance.

### 3.3. Reasons for Not Replacing Missing Teeth

The most frequently reported barrier was financial limitation, especially among older patients and university graduates (*p*  < 0.001). Fear of prosthesis discomfort was also more prevalent among the older age group.

Gender and employment status did not significantly influence the reasons for not seeking replacement (*p*  > 0.05(Tables 2 and 3).

### 3.4. Awareness of Prosthetic Options

Awareness varied across prosthetic types as follows:

Awareness of implants and fixed prostheses was significantly higher among participants with higher education levels (*p*  < 0.001).

Awareness of complete dentures was significantly higher among employed patients (*p*  < 0.001).

No significant differences in awareness were noted between males and females (*p*  > 0.05) or across different age categories for most prosthesis types (*p*  > 0.05), except for removable partial dentures, where age differences were significant (Tables 4 and 5).

**Table 4:** Distributionof patients according to awareness by age and gender.

Variables		Age										Total		Gender			
		<20		20–29		30–39		40–49		>50				Males		Females	
		N.	%	N.	%	N.	%	N.	%	N.	%	N.	%	N.	%	N.	%
Complete denture	Yes	0	0.0	29	16.8	56	32.0	49	28.0	41	23.3	175	87.5	94	81	81	46.29
	No	4	16.0	6	24.0	8	32.0	7	28.0	0	0.0	25	12.5	9	16	16	64.00
Fisher exact	24.192													Chi‐square		2.748	
*p*‐Value	**0.000**													*p*‐Value		0.097	
Fixed partial denture	Yes	1	0.7	24	16.0	53	35.3	40	26.7	32	21.3	150	75.0	75	75	75	50.00
	No	3	6.0	11	22.0	11	22.0	16	32.0	9	18.0	50	25.0	28	22	22	44.00
Fisher exact	8.062													Chi‐square		0.540	
*p*‐Value	0.078													*p*‐Value		0.462	
Dental implant	Yes	3	1.7	32	17.6	58	31.9	50	27.5	39	21.4	182	91.0	94	88	88	48.35
	No	1	5.6	3	16.7	6	33.3	6	33.3	2	11.1	18	9.0	9	9	9	50.00
Fisher exact	2.867													Chi‐square		0.018	
*p*‐Value	0.558													*p*‐Value		0.894	
Removable Partial Denture	Yes	0	0.0	27	18.1	48	32.2	41	27.5	33	22.2	149	74.5	78	71	71	47.65
	No	4	7.9	8	15.7	16	31.4	15	29.4	8	15.7	51	25.5	25	26	26	50.98
Fisher exact	10.195													Chi‐square		0.169	
*p*‐Value	0.032													*p*‐Value		0.681	

*Note:* Bold value shows the significant difference between age and presence or abscence of complete denture.

**Table 5:** Distribution of patients according to awareness by education and employment status.

Variables	Prosthesis status	Education								Employment status					
		Graduated		Secondary		Primary				Employed		Unemployed			
		N.	%	N.	%	N.	%	Chi‐square	*p*‐Value	N	%	N	%	Chi‐square	*p*‐Value
Complete denture	Yes	118	67.43	31	17.71	26	14.86	9.350	**0.009**	132	75.4	43	24.6	6.022	**0.014**
	No	9	36.00	9	36.00	7	28.00			13	52.0	12	48.0		
Fixed partial denture	Yes	97	64.67	29	19.33	24	16.00	0.353	0.838	106	70.7	44	29.3	1.011	0.315
	No	30	60.00	11	22.00	9	18.00			39	78.0	11	22.0		
Dental implants	Yes	119	65.38	37	20.33	26	14.29	7.250	**0.027**	132	72.5	50	27.5	0.001	0.987
	No	8	44.44	3	16.67	7	38.89			13	72.2	5	27.8		
Removable partial denture	Yes	97	65.10	28	18.79	24	16.11	0.717	0.699	113	75.8	36	24.2	3.267	0.071
	No	30	58.82	12	23.53	9	17.65	9.350		32	62.8	19	37.3		

*Note:* Bold values represent statistically significant results (*p* < 0.05) indicating a significant association between the demographic variables and the dependent variables.

### 3.5. Demand and Preferences for Prosthetic Replacement

Overall, 85.5% of participants expressed a demand for tooth replacement, and 46.5% were already wearing some form of prosthesis.

When asked about preferred treatment options:

• 53% preferred dental implants, primarily for aesthetic reasons.
• 26.5% preferred fixed prostheses, often because they do not involve surgery and are considered more accessible.
• 18% preferred removable prostheses, mainly due to lower cost.

Demand for complete dentures increased significantly with age (*p*  < 0.001; (Table 6).

**Table 6:** Distribution of patients according to appliance preferences.

Variables	Total	
	Number	%
*Prosthesis preference*		
Implants	106	53.0
Fixed prosthesis	53	26.5
Removable prosthesis	36	18.0
Refused replacement	5	2.5
*Tooth-borne FPD preference*		
Better esthetic	45	22.5
Cheaper	50	25.0
Not removed	45	22.5
No surgery required	22	11.0
Others	22	11.0
Small size	16	8.0
*Implant preference*		
Better esthetic	95	47.5
Lasts longer	85	42.5
No teeth preparation	20	10
*RPD preference*		
It is the cheaper option	91	45.5
No surgery required	52	26
Others	22	11
Less teeth damage	35	17.5

Abbreviations: FPD, fixed partial denture; RPD, removable partial denture.

## 4. Discussion

The results of this study provide valuable insights into the knowledge, attitudes, and behaviors of patients at Ajman University toward various prosthodontic treatment options for replacing missing teeth. The findings revealed that older patients had a higher incidence of missing teeth, consistent with previous studies indicating that tooth loss increases with age [8, 17, 18]. The gender distribution of partially dentate and edentulous patients in this study, where males had more missing teeth than females, aligns with the findings of Rahman et al. [21] and other studies [6, 10, 28], suggesting that males are less attentive to oral health. However, Ahmed et al. [19] reported contrary results, indicating higher edentulism in females.

Although studies [6, 10] suggest that males may be less attentive to overall oral health, these references primarily address general oral health behaviors rather than prosthetic‐seeking tendencies. In contrast, Mukatash et al. [28] specifically examined prosthetic needs and demands and reported lower motivation among males to replace missing teeth. This distinction supports our finding that male participants in this study not only had more missing teeth but also demonstrated delays in seeking prosthodontic treatment, reflecting both reduced preventive behavior and lower prosthetic motivation.

The lack of a significant correlation between tooth loss and educational level or employment status in this study contrasts with findings from Gupta et al. [17] and Jeyapalan and Krishnan [38], who reported that higher education levels are associated with better oral health and fewer missing teeth. This discrepancy could be due to the high proportion of educated and employed individuals in our sample, potentially masking any underlying correlations. The study also found that females sought prosthodontic treatment earlier than males, which may be attributed to their availability, as most unemployed participants were female. In addition to differences in availability, previous literature suggests that females generally demonstrate greater aesthetic awareness, stronger motivation toward oral health maintenance, and higher dental attendance rates compared with males. These behavioral and psychosocial factors may further explain why female participants in our study sought prosthodontic replacement earlier than males. This is consistent with Prabhu et al. [4] but contrasts with Esan et al. [9] and Jayasinghe et al. [27], who found no significant gender differences in the demand for tooth replacement. Education played a significant role in the promptness of seeking replacement, with educated individuals seeking treatment sooner, as observed in other studies [8, 9].

A notable 85.5% of participants exhibited a positive attitude toward replacing missing teeth, a higher percentage than reported by Jayasinghe et al. [27] and Mukatash et al. [28]. Financial constraints were the primary barrier to seeking treatment, like previous findings [7, 17, 18]. Other barriers included time constraints, perceived discomfort, and the belief that replacement was unnecessary. The presence of previous prostheses was higher among patients with primary education, a finding that might be influenced by the specific nature of our sample, which consisted of patients attending the university’s clinics. This contrasts with the results of other studies that found higher replacement rates among more educated patients [4, 8]. The higher incidence of edentulism without replacement among older participants may be due to their adaptation to the situation, as opposed to younger patients who might be more inclined to seek replacement.

Awareness of prosthetic options was reasonable at 74%, though lower than Alshehri et al., who reported 87% awareness in a similar sample [7]. Awareness was highest for removable prostheses and lowest for implants, consistent with previous studies [11, 15, 20, 22, 23, 25–27]. Education significantly influenced awareness, with higher awareness among educated individuals, as reported by others [3, 4, 7, 19]. However, Jayasinghe et al. [27] found no significant relationship between education and awareness. The preference for dental implants as the most favored replacement option, followed by fixed and removable dentures, supports earlier findings by Mukatash et al. [28] and Srinagar and Zargar [22]. The choice of implants was driven by their superior aesthetic qualities, while cost‐effectiveness influenced the selection of fixed and removable dentures.

The findings of this study may also be interpreted within the socioeconomic and cultural context of the United Arab Emirates. In this region, females generally demonstrate higher engagement in routine dental care and place stronger emphasis on aesthetics, which may contribute to their earlier seeking of prosthodontic treatment. Moreover, university dental clinics in the UAE provide affordable care, making them particularly accessible to unemployed individuals, many of whom in our sample were female. Financial barriers also remain a prominent factor in the UAE dental landscape, which may explain why cost was the most frequently cited reason for delayed replacement among participants. These contextual factors help explain the patterns observed in our study and highlight the importance of local socioeconomic dynamics in shaping oral health behaviors.

Fisher’s exact test was applied in instances where the expected frequencies in one or more contingency‐table cells were very small. Under such conditions, the assumptions of the chi‐square test are violated, reducing its reliability. Fisher’s exact test provides an exact probability estimate and is therefore the more accurate and appropriate method for analyzing associations when cell counts fall below five or when sample distributions are uneven.

The study’s limitations include data collection from a single location, a restricted number of patients, uneven age categories, and uneven distribution across different age groups and genders.

This study did not analyze associations between clinical status (number of missing teeth, type of edentulism, or presence of existing prostheses) and patient responses, which should be addressed in future research.

Despite these limitations, the study provides valuable insights into the prosthodontic needs and preferences of patients at Ajman University, highlighting the importance of educational and financial factors in shaping their attitudes and behaviors toward tooth replacement. Thus, this study proves the need for targeted educational programs to enhance patient awareness and address financial barriers to improve the uptake of prosthodontic treatments. Further research with a larger and more diverse sample is recommended to validate these findings and develop more effective interventions.

## 5. Conclusion

This study reveals a significant level of awareness and generally positive attitudes toward prosthodontic treatment among patients attending Ajman University. The independent variables assessed—age, gender, education, and employment status—showed distinct impacts on the study outcomes. Older patients, particularly males, demonstrated a higher incidence of tooth loss, while females sought prosthodontic replacement earlier, reflecting differences in health‐seeking behavior. Education emerged as a key determinant in both the promptness of seeking treatment and the level of awareness of prosthetic options, whereas employment status influenced certain aspects of awareness, particularly regarding complete dentures. Financial constraints, however, remained the primary barrier across demographic groups. Dental implants were the most preferred option, attributed to their aesthetic advantages, though cost considerations led some patients to favor fixed or removable dentures.

These findings highlight the need for targeted educational initiatives and financial support programs that address demographic disparities and improve access to prosthodontic care. Further research with broader and more diverse populations is recommended to validate and expand upon these results.

## Conflicts of Interest

The authors declare no conflicts of interest.

## Funding

No funding was received for this manuscript.

## Data Availability

The data that support the findings of this study are available upon request from the corresponding author. The data are not publicly available due to privacy or ethical restrictions.
